# Modified Citrus Pectin Reduces Galectin-3 Expression and Disease Severity in Experimental Acute Kidney Injury

**DOI:** 10.1371/journal.pone.0018683

**Published:** 2011-04-08

**Authors:** Maria Kolatsi-Joannou, Karen L. Price, Paul J. Winyard, David A. Long

**Affiliations:** Nephro-Urology Unit, UCL Institute of Child Health, London, United Kingdom; Universidade de Sao Paulo, Brazil

## Abstract

Galectin-3 is a β-galactoside binding lectin with roles in diverse processes including proliferation, apoptosis, inflammation and fibrosis which are dependent on different domains of the molecule and subcellular distribution. Although galectin-3 is known to be upregulated in acute kidney injury, the relative importance of its different domains and functions are poorly understood in the underlying pathogenesis. Therefore we experimentally modulated galectin-3 in folic acid (FA)-induced acute kidney injury utilising modified citrus pectin (MCP), a derivative of pectin which can bind to the galectin-3 carbohydrate recognition domain thereby predominantly antagonising functions linked to this role. Mice were pre-treated with normal or 1% MCP-supplemented drinking water one week before FA injection. During the initial injury phase, all FA-treated mice lost weight whilst their kidneys enlarged secondary to the renal insult; these gross changes were significantly lessened in the MCP group but this was not associated with significant changes in galectin-3 expression. At a histological level, MCP clearly reduced renal cell proliferation but did not affect apoptosis. Later, during the recovery phase at two weeks, MCP-treated mice demonstrated reduced galectin-3 in association with decreased renal fibrosis, macrophages, pro-inflammatory cytokine expression and apoptosis. Other renal galectins, galectin-1 and -9, were unchanged. Our data indicates that MCP is protective in experimental nephropathy with modulation of early proliferation and later galectin-3 expression, apoptosis and fibrosis. This raises the possibility that MCP may be a novel strategy to reduce renal injury in the long term, perhaps via carbohydrate binding-related functions of galectin-3.

## Introduction

Galectins are low molecular weight, calcium-independent, β-galactoside-binding lectins [1]. Galectin-3 is a multi-domain molecule which includes an N-terminal proline-rich domain and a C-terminal carbohydrate recognition domain essential for binding simple β-galactosides such as lactosamine and Galβ1-4GlcNAc; and for higher affinity binding to polylactosamine chains [2]. Galectin-3 plays a key role in several intracellular physiological and pathological processes including proliferation and apoptosis, via carbohydrate-independent mechanisms [3]–[5]. In addition, galectin-3 is involved in modulation of cell-cell interactions and inflammation, predominately through extracellular carbohydrate binding functions [6]–[9]. In the kidney, galectin-3 is strongly expressed in the ureteric bud and its derivatives, the collecting ducts, in normal development and the mature organ [8], [10]. Lower levels are also sometimes observed in mature tubules [11] but the lectin is expressed in a more widespread distribution in models of acute kidney damage such as ischemia-reperfusion injury or high-dose folic acid (FA) treatment [12]. In this latter model, FA initially undergoes glomerular filtration following systemic injection, and precipitates in the tubules which become damaged with a loss of epithelial cell integrity due to necrosis and apoptosis [13], [14]. After two days, the majority of the tubules show regenerative changes as new cells proliferate and migrate to repair the denuded areas of the tubule [15]. However, over the next two weeks there is incomplete healing in some areas of the kidney, as evidenced by patchy interstitial fibrosis, loss of peritubular capillaries and inflammation with macrophage infiltration [16], [17]. In the FA model, galectin-3 expression is initially observed in both proximal and distal tubules, and thick ascending limbs as well as collecting ducts; later, it is detectable in macrophages, particularly in areas of inflammation [12]. Several lines of evidence suggest that galectin-3 is beneficial in experimental kidney diseases such as polycystic kidney disease [18], nephrotoxic nephritis [19] and unilateral ureteric obstruction (UUO) [11], but its functional importance in FA-induced acute kidney injury is unknown. Therefore, we utilised this model and modulated galectin-3 levels using modified citrus pectin (MCP), a derivative of pectin; which is a soluble dietary fibre found in the peel and pulp of citrus fruits [20]. MCP contains fragments of the original pectin molecule, including rhamnogalacturonan 1 regions which contain galactan side-chains [21] which bind to the carbohydrate recognition domain of galectin-3 [22], [23], hence modulating galectin-3 bioactivity by altering extracellular functions such as cell-cell interactions and inflammation.

## Methods

### Experimental Strategy

**Figure 1:**
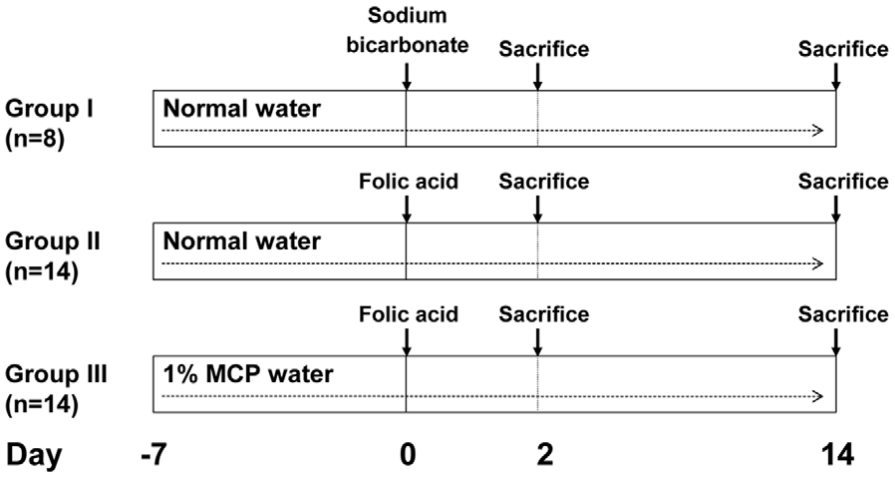
Experimental groups I-III.

Reagents were obtained from Sigma Chemical Company (Poole, UK) unless otherwise stated. Eight week-old male C57Bl/6J mice (Charles River Laboratories, Margate, UK), of average weight 25 g were used in procedures approved by the UCL local ethics committee and the UK Home Office (project licence PPL 70/6627). The three main experimental groups are depicted in Figure 1. Group I (n = 8), the ‘sham controls’, were provided with normal drinking water *ad libitium* throughout, injected with intraperitoneal (IP) sodium bicarbonate (NaHCO_3_, 0.2 ml, 0.3 M; the vehicle used for FA administration) at Day 0; and were killed either at Day 2 (n = 4) or Day 14 (n = 4). Groups II and III were used to induce FA-nephropathy. Group II (n = 14), were also provided with normal drinking water throughout but were injected on Day 0 with 240 µg/g body weight FA, a dose which reliably caused ‘acute tubular necrosis’ in all mice, with a low mortality rate (less than 5%) over 14 days observation [16], [17]. Group III (n = 14), were administered 1% MCP (Pectasol, EcoNugenics, Santa Rosa, CA) in the drinking water for seven days before the IP FA and then continued on MCP throughout the protocol. This dose of MCP previously led to effective galectin-3 blockade and was non-toxic in murine cancer studies examining parameters such as tumour growth, angiogenesis and spontaneous metastasis [24]. Lower doses of MCP also prevent galectin-3 mediated functions in-vitro including chemotaxis and cell adhesion [24], [25], but there is no data showing this would be replicated in-vivo. Experiments were also performed to ensure that pectin itself did not have adverse effects. In both Groups II and III, one mouse died spontaneously at Day 1 and the remainder were killed at either Day 2 or Day 14 of the protocol. All of the assessments were made blinded to experimental groups.

### Histology and Blood Analyses

Body and kidney weights were measured and right-sided kidneys used for histology and left-sided kidneys for biochemical studies. Blood was collected by exsanguination and commercially available assay kits, validated in mice, were used to assess creatinine (Cusabio, Newark, Delaware) and blood urea nitrogen (BUN) (BioAssay Systems, Hayward, CA). Kidneys were fixed in 4% paraformaldehyde, embedded in wax, then five µm sections were cut, dewaxed and rehydrated for immunohistochemistry as described [17] for primary antibodies: rabbit anti-human galectin-3 (Santa Cruz Biotechnology, Santa Cruz, CA); rat anti-mouse macrophage marker (F4/80; Serotec, Raleigh, NC), rat anti-mouse neutrophil (MCA771G; Serotec), goat anti-human collagen I (Southern Biotech, Birmingham, AL), goat anti-human collagen III (Southern Biotech), and mouse anti-human proliferating cell nuclear antigen (PCNA, BD Biosciences, Oxford, UK). Negative controls consisted of omission of primary antibodies or substitution with preimmune serum. Some sections were stained with either periodic acid Schiff reagent (PAS) and/or haematoxylin. For collagen I, collagen III, and macrophage staining photomicrographs of 20 sequential fields using an ×20 objective were taken and the area of the kidney cortex containing positive immuno-reactivity was analysed as a percentage of the whole image using Image J software (http://rsbweb.nih.gov.ij/) [16]. The number of positive galectin-3 interstitial cells was also determined in 20 random fields from the renal cortex using ×20 objective. For PCNA and neutrophil staining, the numbers of positive cells were counted in both 20 random fields from the renal cortex and 50 glomeruli for each kidney using ×20 and ×40 objectives respectively. Detection of apoptosis was performed by TUNEL staining as previously described [26]. For each kidney, the numbers of positive cells were counted in both 20 random fields from the renal cortex and 50 sequential glomeruli.

### Real-Time Polymerase Chain Reaction (RT-PCR)

RNA was extracted using the RNeasy kit (Qiagen, Crawley, West Sussex, UK) from whole kidneys of animals and 500 ng used to prepare cDNA. Quantitative RT-PCR was performed for both fibrotic genes (α-smooth muscle actin (α-SMA), collagen I, collagen III, fibronectin, galectin-1, galectin-3, galectin-9 and transforming growth factor β1 (TGFβ1)) and cytokines (interleukin-1β (IL-1β), interleukin-6 (IL-6), monocyte chemoattractant protein-1 (MCP-1) and tumour necrosis factor-α (TNF-α)) using previously described methods [27] with hypoxanthine-guanine phosphoribosyltransferase (HPRT) as a house-keeping gene. Primer details are available on request.

### Western blotting

Western blotting was performed on 100 µg of protein prepared from whole kidney tissue as previously described [17]. Blots were incubated with rabbit anti-human galectin-3 antibody (Santa Cruz Biotechnology) and bands detected by chemiluminescence. Blots were stripped and then reprobed for GAPDH (Abcam, Cambridge, UK) as a housekeeping gene. Band intensity was measured by densitometry.

### Statistics

Data were presented as means ± standard error of the mean (SEM). Differences in the various parameters between groups were evaluated by Mann-Whitney *U*-test (SPSS, Chicago, IL). Statistical significance was accepted at p<0.05.

## Results

### Galectin-3 expression is more widespread in FA nephropathy

**Figure 2:**
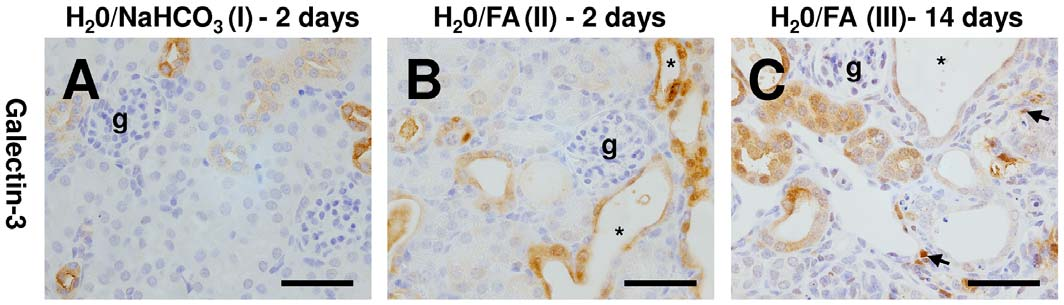
Galectin-3 expression in FA nephropathy. A. Sham control kidneys contained positive galectin-3 expression primarily in collecting ducts. B. Two days after FA administration, prominent galectin-3 expression was observed in dilated tubules (indicated by *, g  =  glomerulus). This was maintained in the recovery phase of FA nephropathy after 14 days, but in addition positive galectin-3 was observed in infiltrating cells in fibrotic areas (arrows, C). Bars are 50 µm.

Sham control kidneys contained positive galectin-3 expression in collecting ducts (Figure 2A). Two days after FA administration, widespread galectin-3 expression was observed in diverse subsets of tubules including dilated proximal tubules as well as collecting ducts (Figure 2B). This expanded distribution was maintained in the recovery phase of FA nephropathy after 14 days and in addition galectin-3 positive cells were observed in fibrotic areas consistent with macrophage localisation (Figure 2C).

### MCP administration reduces galectin-3 upregulation in the recovery phase of FA nephropathy

**Figure 3:**
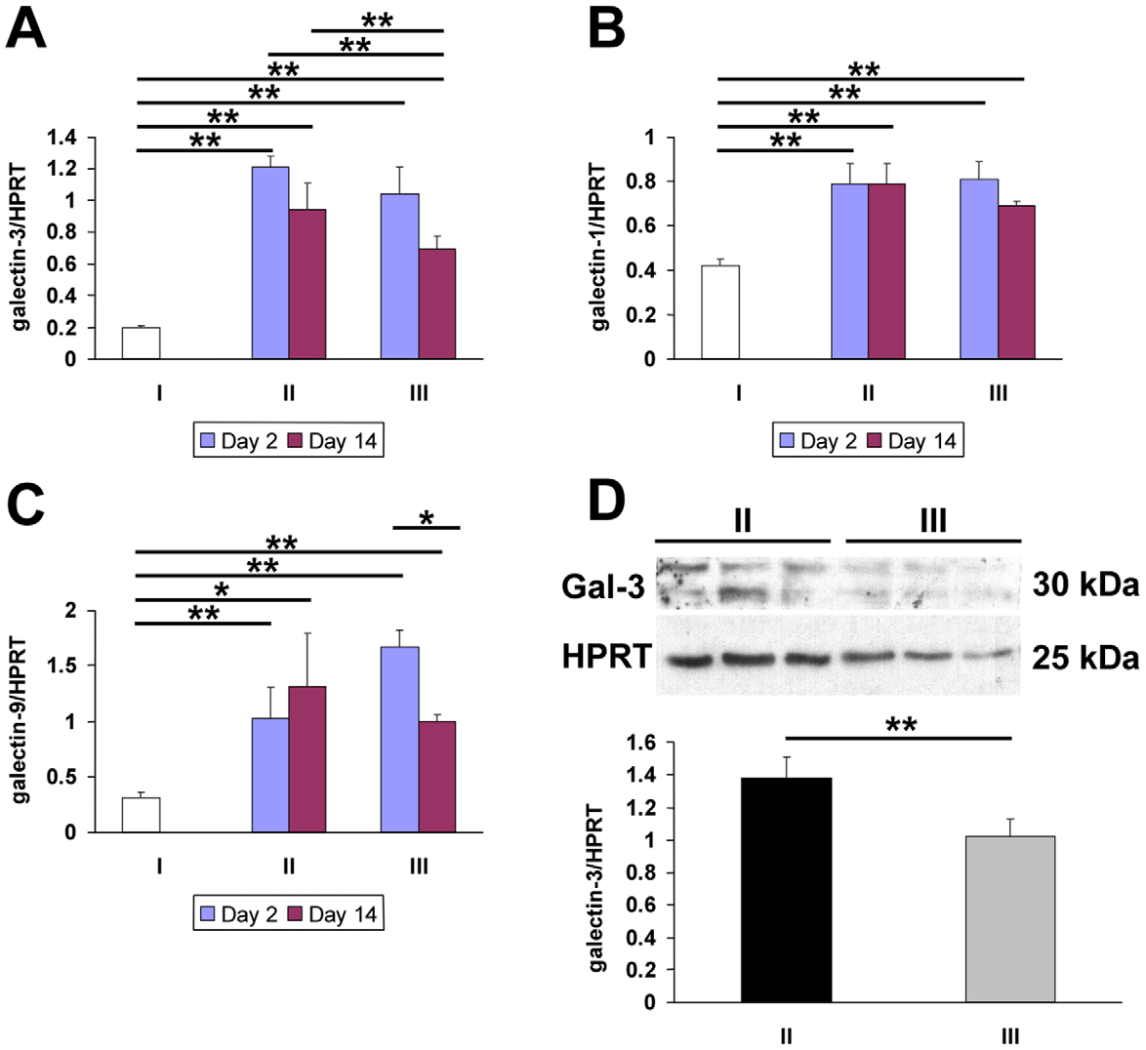
Effect of MCP on galectin expression in FA nephropathy. Quantitative RT-PCR for renal galectin-3 (A), galectin-1 (B) and galectin-9 (C) on kidney RNA from animals in Group I (water throughout, NaHCO_3_ injection), Group II (water throughout, FA injection) and Group III (MCP throughout and FA injection), two and fourteen days after FA administration (n = 4 in Group I, 6–7 at each time-point in Group II and Group III). All galectins were significantly upregulated in FA-treated animals at both time-points, but MCP only diminished galectin-3 levels fourteen days after induction of FA nephropathy. (D) Western blotting for galectin-3 in kidney samples from Groups II and III collected 14 days after induction of FA nephropathy. Following densitometry analysis (n = 6–7 in each group), galectin-3 protein levels were shown to be significantly decreased in FA animals administered MCP. ** indicates p<0.01 between Groups while * indicates p<0.05 between Groups.

To assess the effect of MCP on galectin-3 levels, we used quantitative RT-PCR and Western blotting. In agreement with our immunostaining observations, kidneys of FA-exposed animals (Group II) contained significantly higher galectin-3 mRNA levels than sham controls (Group I) at both experimental day 2 and 14 (Figure 3A). Administration of MCP (Group III) did not significantly alter galectin-3 mRNA levels at 2 days but there was a statistically significant reduction at day 14 (Figure 3A). This reduction was confirmed at the protein level at day 14: a galectin-3 doublet was detected at the expected size of around 30 kDa which was significantly decreased in FA-animals administered MCP (Figure 3D). At this time-point the number of galectin-3 positive interstitial cells was also significantly decreased in MCP FA-treated mice compared to FA-animals drinking normal water (13.6±1.2 versus 23.8±3.1 positive interstitial cells/field respectively, p<0.01). Since galectin-1 and galectin-9 are also expressed in the kidney and might potentially substitute for some of the roles of galectin-3, we also assessed their mRNA levels following FA and pectin. Both galectin-1 and galectin-9 mRNA levels were upregulated in FA animals compared to sham controls in both the acute and recovery phases, but these increases were not significantly altered by MCP (Figure 3B–C).

### MCP administration reduces proliferation in the acute phase of FA nephropathy

**Figure 4:**
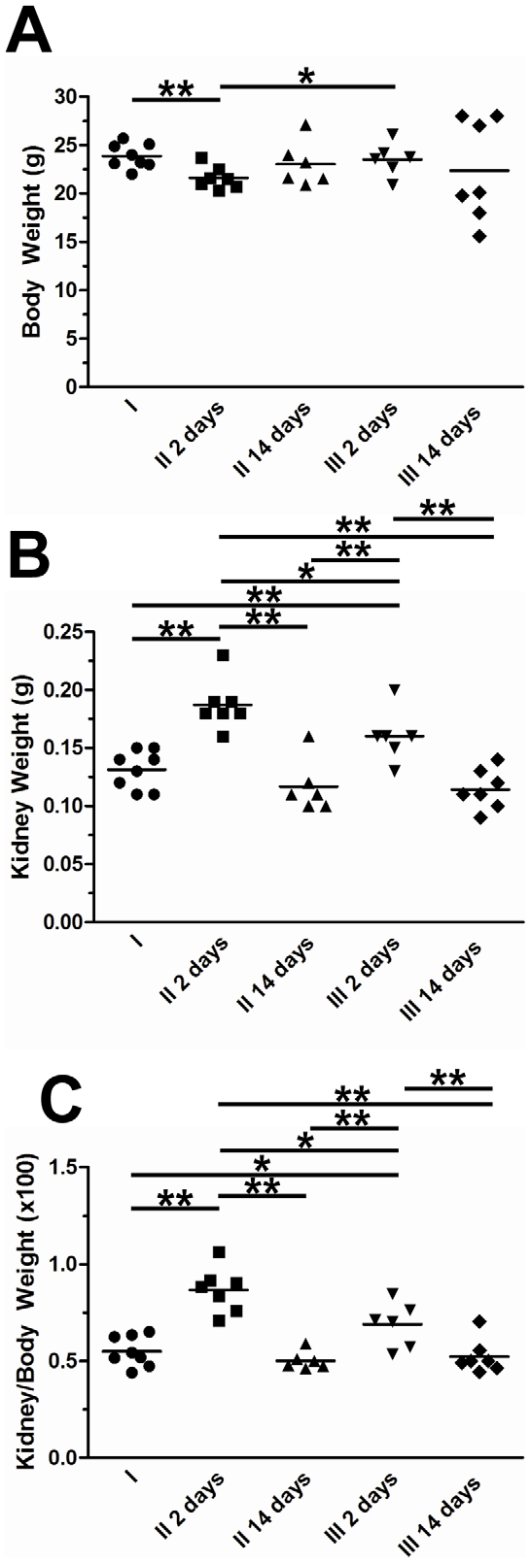
Assessment of kidney and body weights. Body (A), kidney (B) and kidney/body weights (C) were assessed in Group I (normal water throughout, injected with vehicle NaHCO_3_ as sham control), Group II (normal water throughout, injected with FA) and Group III (normal water supplemented with 1% MCP for 7 days prior to injection with FA, and continued thereafter). Two days after FA, animals drinking normal water had elevated kidney weights, increased kidney/body weights and decreased body weights compared to sham controls. MCP administration prevented the loss in body weight at this time-point with levels similar to sham controls. Kidney weights and kidney/body weights in MCP FA-exposed animals two days after FA exposure remained higher than sham controls but were significantly reduced compared to FA animals drinking normal water. By Day 14 of the protocol, the initial swelling in FA-exposed kidneys subsided, with kidney, body and kidney/body weight ratios returning to those observed in sham controls; administration of MCP did not alter kidney/body weight ratios at this time-point. ** indicates p<0.01 between Groups while * indicates p<0.05 between Groups.

**Figure 5:**
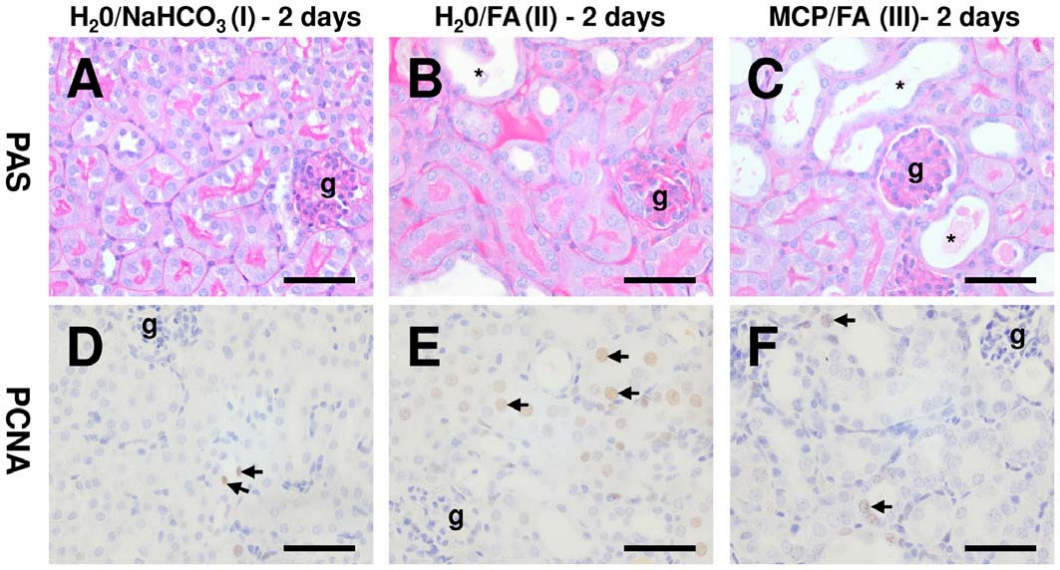
Kidney histology two days after FA exposure. A–C. PAS staining on a representative sham control and FA kidneys after 2 days. Marked kidney damage was noted in all animals administered FA, with flattened proximal and distal tubule epithelia with casts in tubule lumens (indicated by *, g  =  glomerulus). There was no observed difference between FA animals administered normal drinking water (B) or those supplemented with 1% modified citrus pectin (C). D–F. Assessment of proliferation by PCNA immunostaining. D. Occasional positive cells (brown signal, arrows indicate some of these positive cells) were observed in sham controls. E. Kidneys of animals injected with FA showed markedly increased numbers of PCNA positive cells predominately in tubular epithelium. Kidneys from FA animals provided with MCP in the drinking water had significantly decreased numbers of PCNA positive cells (F, p<0.05). Bars are 50 µm.

All mice treated with FA became unwell and developed acute renal injury within two days, with increases in serum creatinine of 7–8 fold and in BUN of 9–10 fold (Table 1). FA animals drinking normal water lost just under 10% of their body weight and had significantly enlarged kidneys compared to sham controls (Figure 4, Table 1). Histological examination revealed acute kidney damage in all animals administered FA, with flattened proximal and distal tubule epithelia and casts in tubule lumens (Figure 5A–C). There was also increased cell proliferation as evidenced by more frequent mitotic figures and significantly elevated numbers of cells expressing the proliferation marker PCNA when compared to sham controls (Figure 5D–F, Table 2). There were also more apoptotic cells (Table 2). There were virtually no proliferating or apoptotic cells observed in the glomeruli in any of the experimental groups, confirming our prior studies that FA primarily induces tubular damage [16], [17]. We also assessed the expression of the fibrosis-associated genes collagen I and III using quantitative immunohistochemistry: both collagens were significantly upregulated in the kidneys of FA-exposed animals versus sham controls. Unsurprisingly, since there is little macrophage infiltration at this stage of the disease [17], we did not observe any changes in the area of the kidney containing F4/80 positive cells between FA-exposed and sham control animals (Table 2).

**Table 1:** Kidney weights and renal excretory function.

	Group IH_2_0▸NaHCO_3_	Group IIH_2_0▸FA		Group IIIMCP▸FA	
	(n = 8)	Day 2 (n = 7)	Day 14 (n = 6)	Day 2 (n = 6)	Day 14 (n = 7)
**Body weight (g)**	23.8±0.4	21.6±0.4^a^	23.1±0.9	23.6±0.7^d^	22.4±2.0
**Kidney weight (g)**	0.13±0.01	0.19±0.01^a^	0.12±0.01^c^	0.16±0.01^a,d,e^	0.11±0.01^c,f^
**Kidney/body weight (x100)**	0.55±0.03	0.87±0.04^a^	0.50±0.02^c^	0.69±0.05^b,d,e^	0.52±0.03^c,f^
**Serum creatinine** **(µM)**	22±1	147±15^ a^	40±1^a,c^	157±8^a,e^	37±2^a,c,f^
**BUN (mM)**	7±1	72±17^a^	18±2^a,c^	62±4^a,e^	16±1^a,c,f^

Modified citrus pectin (MCP) or normal drinking water was administered seven days prior to the injection of either FA or vehicle only (NaHCO_3_) at Day 0. All data are given as mean ± SEM. a  =  p<0.01 compared to Group I, b =  p<0.05 compared to Group I, c =  p<0.01 compared to Group II at day 2, d = p<0.05 compared to Group II at day 2, e =  p<0.01 compared to Group II at day 14, f =  p<0.01 compared to Group III at day 2.

**Table 2:** Histological parameters.

	Group I H_2_0▸NaHCO_3_	Group IIH_2_0▸FA		Group IIIMCP▸FA	
	(n = 8)	Day 2 (n = 7)	Day 14 (n = 6)	Day 2 (n = 6)	Day 14 (n = 7)
**Proliferating cells (number per field)**	0.9±0.3	17.9±3.3^a^	3.8±0.4^a,b^	7.2±2.8^a,c^	5.1±1.3^a,b^
**Apoptotic cells (number per fields)**	0.7±0.1	2.7±0.6^a^	4.3±1.4^a,b^	1.8±0.6^a,d^	2.8±0.2^a,d^
**Collagen I area (%)**	0.5±0.1	2.5±0.7^a^	1.3±0.2^a^	2.3±0.4^a^	0.5±0.1^b,e,f^
**Collagen III area (%)**	1.2±0.1	3.0±0.3^a^	2.6±0.3^a^	2.2±0.2^a^	2.2±0.3^b^
**Macrophage area (%)**	0.4±0.1	0.2±0.1^a^	6.5±1.0^b^	0.3±0.1^d^	3.8±0.5^a,b,e,f^

Modified citrus pectin (MCP) or normal drinking water was administered seven days prior to the injection of either FA or vehicle only (NaHCO_3_) at Day 0. All data are given as mean ± SEM. a  =  p<0.01 compared to Group I; b  =  p<0.01 compared to Group II data at Day 2; c =  p<0.05 compared to Group II data at day 2; d  =  p<0.01 compared to Group II data at Day 14; e  =  p<0.05 compared to Group II data at Day 14; f  =  p<0.01 compared to Group III data at Day 2.

FA also caused severely impaired kidney function in MCP-maintained mice, with increases in serum creatinine and BUN comparable to those given normal drinking water. Clinically, however, the MCP mice had reduced severity of disease: they had preserved body weight (mean 23.6 g), which is similar to sham controls (Figure 4, Table 1), whilst kidney weights (0.16 g) were higher than shams but significantly reduced compared to those exposed to FA but maintained on water alone (Figure 4, Table 1). Histologically, MCP administration significantly reduced the number of proliferating cells in FA-treated animals (water/FA 17.9±3.3; MCP/FA 7.2±2.8) but did not significantly alter levels of apoptosis (water/FA 2.7±0.6, MCP/FA 1.8±0.6). MCP administration did not alter collagen I or III expression (Table 2).

### MCP reduces fibrosis and apoptosis after 14 days

Mice are in the recovery phase by day 14, and we observed body and kidney weight similar to sham values in both water and MCP-maintained animals (Figure 4, Table 1). The whole of the kidney does not return to normal, however, and depressed areas on the kidney surface were detectable in all FA animals, indicating underlying tissue fibrosis. Blood creatinine and BUN levels improved from the acute phase; but both remained significantly increased compared to sham controls (Table 1; creatinine elevated 1.7–1.9 fold; BUN up 2.1–2.3x). Levels were marginally higher in those drinking normal rather than MCP-supplemented water, but this was not statistically significant (Table 1).

**Figure 6:**
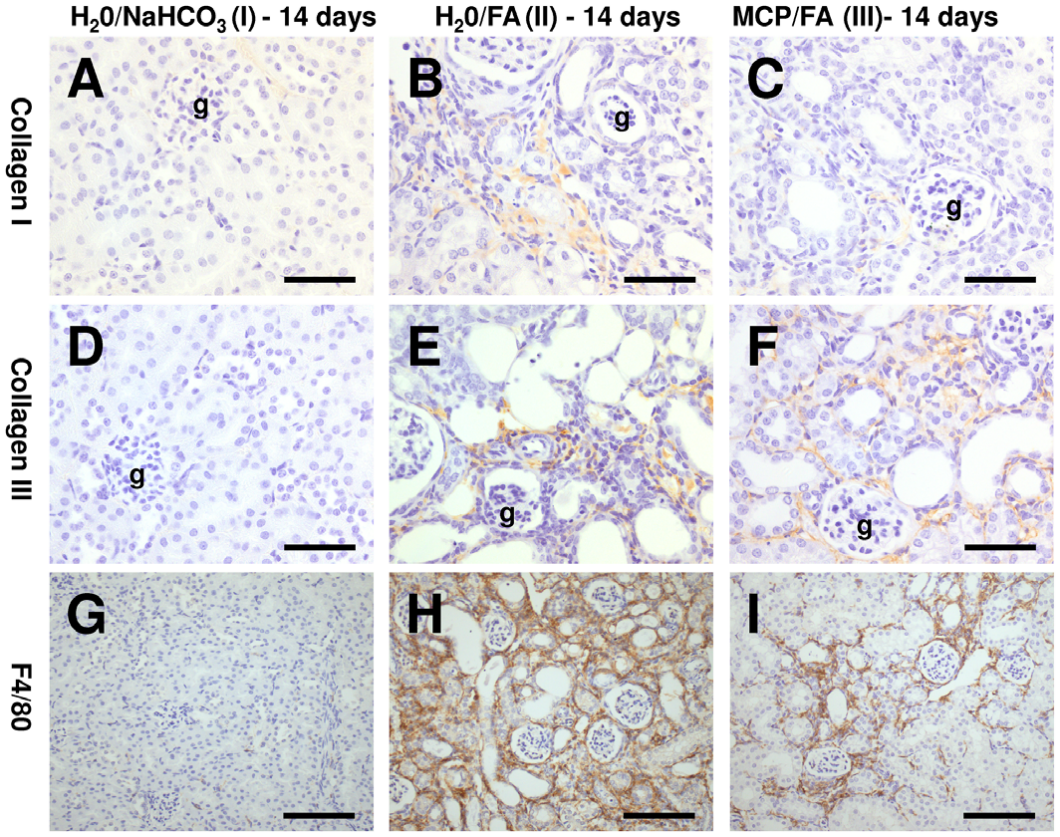
Histological observations fourteen days after FA nephropathy. Immunohistochemistry with collagen I (A–C) and III (D–F) demonstrated patchy fibrotic areas 14 days following FA administration (brown signals, g =  glomerulus) and quantification revealed a marked increase compared to sham controls. Collagen I staining, but not collagen III was reduced in FA-animals administered MCP in their drinking water compared to those drinking normal water. The area of the kidney containing macrophages was increased in animals exposed to FA versus sham controls as assessed by F4/80 staining (G–I) and this increase was attenuated in MCP FA mice. Bars are 50 µm in A–F and 100 µm in G–I.

Histologically, in non-fibrotic areas, most of the cortex appeared normal by 2 weeks after the FA. More cells positive for PCNA were still observed (Table 2) than sham controls but there was no difference between animals drinking unsupplemented and 1% MCP water. Levels of apoptosis were greater than during the acute phase, suggestive of remodeling and deletion of unwanted cells from the regenerative stage. There was a striking difference between the water and MCP-maintained groups, with much more apoptosis in the former (p<0.01, Table 2). In fibrotic areas, collagen I and III deposition and infiltration of inflammatory cells were clearly visible using immunohistochemistry (Figure 6, Table 2); intriguingly, collagen I staining and numbers of macrophages were significantly reduced in the MCP group, but collagen III and numbers of neutrophils in the interstitium (9.6±2.5 and 7.3±1.4 cells/field in FA-animals drinking normal and MCP water respectively) were unchanged.

**Figure 7:**
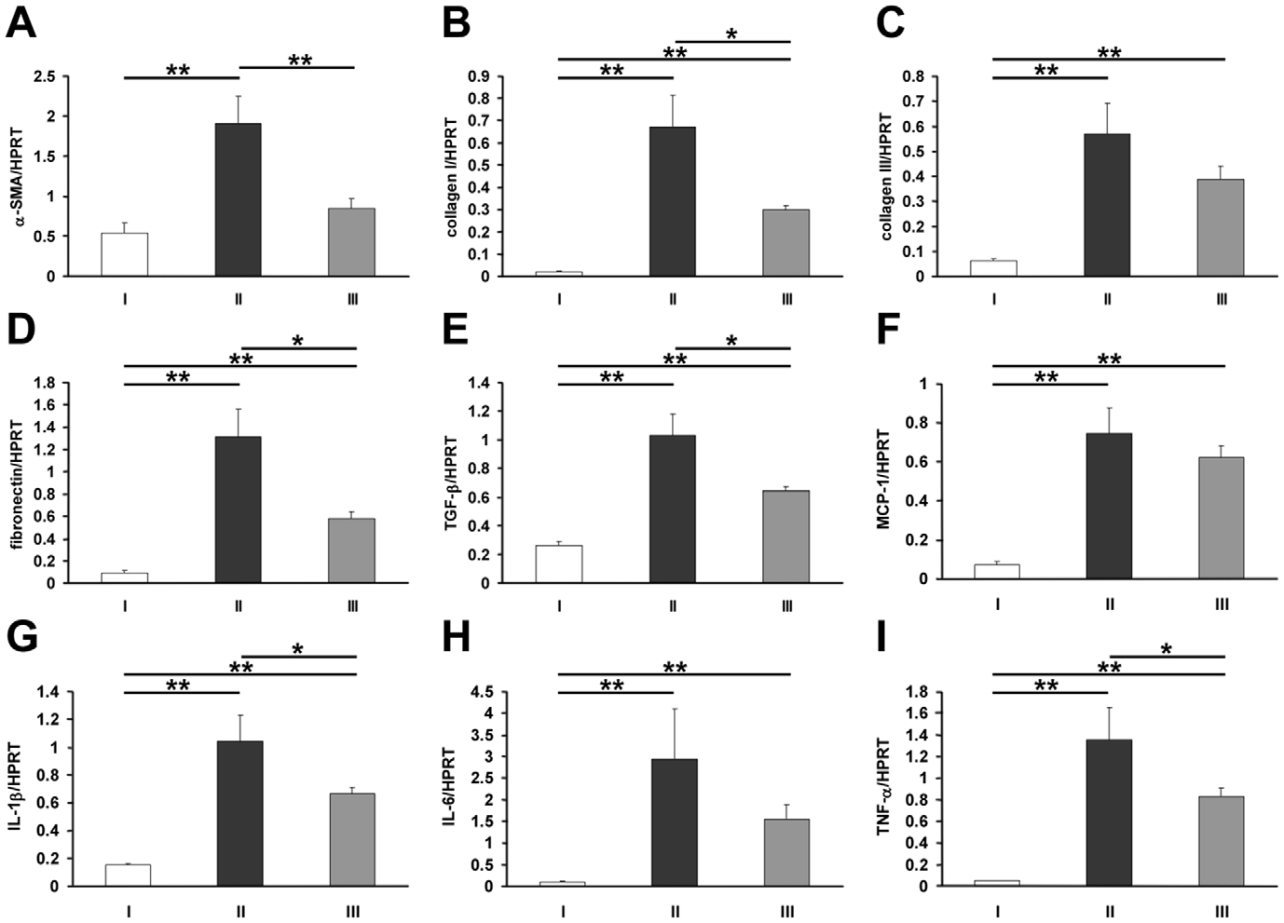
Renal gene expression of fibrotic genes and cytokines. Quantitative RT-PCR for renal α-SMA (A), collagen I (B), collagen III (C), fibronectin (D), TGFβ-1 (E), MCP-1 (F), IL-1β (G), IL-6 (H) and TNF-α (I) on kidney RNA from animals in Group I (water throughout, NaHCO_3_ injection), Group II (water throughout, FA injection) and Group III (MCP throughout and FA injection), two weeks after FA administration (n = 4 in Group I, 6 in Group II and 7 in Group III). All genes were significantly upregulated in FA nephropathy compared to sham controls, but this was attenuated when MCP was provided in the drinking water, except for collagen III, MCP-1 and IL-6. ** indicates p<0.01 while * indicates p<0.05.

We sought quantification by real-time RT-PCR of the collagens and other fibrotic markers including α-SMA, fibronectin, and TGFβ1. There were increased levels of all of these genes in the kidneys of FA-exposed animals compared to sham controls (Figure 7A–E). MCP caused significant attenuation of the increases for most of these fibrotic markers (α-SMA p<0.01; collagen I, fibronectin and TGFβ1 p<0.05); but again did not alter collagen III (p = 0.18). We also examined a selection of cytokines previously shown to be involved in the inflammatory responses in acute kidney disease [28]. FA-exposed kidneys contained elevated levels of the cytokines IL-1β, IL-6, MCP-1, and TNF-α compared to sham controls (Figure 7F–I). MCP led to significantly decreased levels of IL-1β and TNF-α, p<0.05); MCP-1 and IL-6 were both lower but this did not reach statistical significance.

### MCP has no effects in normal healthy animals

One potential explanation for the MCP-associated benefits could be that pectin simply makes the mice drink more, thus they are better hydrated at the time of FA-induced injury and suffer less renal consequences. To address this, we also assessed whether MCP has any effects on mice injected with vehicle (sodium bicarbonate): animals were given water supplemented with MCP for 7 days, then injected with sodium bicarbonate and continued on MCP before analysis two weeks later and compared to Group I. Administration of MCP did not alter water intake, body or kidney weight, renal function, or induce any changes in renal histology as assessed by fibrotic, inflammatory and proliferative markers (data not shown).

## Discussion

In our study, we investigated roles of galectin-3 in the FA model of acute kidney injury using the natural inhibitor MCP. Our principal findings were that MCP reduced acute renal enlargement and proliferative responses in the initial phases of FA nephropathy, then decreased renal apoptosis, inflammation and fibrosis in the later phase. MCP did not alter galectin-3 initially, but the latter effects were associated with significantly decreased galectin-3 levels, with no change in other renal galectins. This data raises the possibility that modulation of galectin-3 may be a novel strategy to reduce acute renal injury. Indeed, Fernandes Bertocchi and colleagues [29] demonstrated acute kidney damage induced by ischemia reperfusion was attenuated in mice lacking galectin-3 with an improvement in blood urea nitrogen 6 and 24 hours after the initial insult, but no difference at later time-points. Structurally, the knock-out animals presented with less acute tubular necrosis and a more prominent tubular regeneration when compared with controls and an improvement in inflammation [29].

MCP is a derivative of pectin; a soluble dietary fibre found in the peel and pulp of citrus fruits [20], and has inhibitory effects on the progression of several animal models of cancer [24], [30]. MCP is rich in β-galactoside residues and binds to the carbohydrate recognition domain of galectin-3 [22], [23] thereby impairing the lectin’s carbohydrate binding-related functions. Therefore, it could be postulated that if MCP acted only through galectin-3 in FA nephropathy it would not have any effect on intracellular actions including proliferation and apoptosis, while modulating extracellular functions such as inflammation. This proved not the case; MCP treatment reduced tubular proliferation two days following FA administration with no differences in galectin-3 expression. Most studies have only considered the effects of MCP in relation to galectin-3, but many other pathways could modulate proliferation here, such as MAP kinase activation [31]. One could speculate that an alternative explanation is that it is not just galectin-3 levels but also bioavailability that should be considered. It is possible that similar levels of galectin-3 have less biological effects when MCP is present because its carbohydrate binding roles will be abrogated. This cannot be measured in-vivo at present, but in-vitro studies have shown that both cell migration [24] and agglutination [32] are diminished in the presence of MCP when induced with similar concentrations of galectin-3. The reduced proliferation could also suggest renal recovery is slower in MCP-treated mice or alternatively that MCP may protect the kidney against structural injury. This second explanation is supported by the fact MCP mice have preserved body weight and their kidneys did not exhibit such acute gross swelling as those exposed to FA but maintained on water alone.

MCP decreased renal mRNA and protein levels of galectin-3 at 14 days after FA injection, in concert with significantly improved renal fibrosis as assessed by reduced expression of multiple fibrotic genes. MCP had no effect on galectin-1 and galectin-9, which are also expressed in the kidney [33], [34], suggesting these effects do not result from MCP interactions with other galectins. The direct correlation of less galectin-3 with less renal injury is consistent with studies by Henderson and colleagues [35] wherein mice lacking galectin-3 have less fibrosis and decreased collagen and α-SMA expression seven days after UUO. In contrast, a recent paper reported that fibrosis severity was increased by day 14 post UUO in galectin-3 deficient mice [11]. These observations may not relate directly to our model, however, because the pathology of chronic UUO is very different to FA-induced injury and both of these studies focussed on knock-out mice where both carbohydrate binding-dependent and -independent functions are abrogated.

The growth factor TGF-β plays a key role in the progression of renal fibrosis by promoting myofibroblastic differentiation [36] and galectin-3 has been implicated in this type of differentiation and extracellular matrix production in hepatic stellate cells [37]. MCP reduced TGF-β mRNA here, which may have contributed to reduced myofibroblast formation as evidenced by decreased α-SMA levels. An unexpected finding was the lack of significant differences in collagen III expression with MCP, which contrasts with the other profibrotic factors examined. A correlation between galectin-3 and collagen I, but not with collagen III, was recently reported by Okamura and colleagues [11]: they noted that lack of galectin-3 led to a relative fall in collagen I but not III over time in UUO. Sharma *et al*. also observed differential effects of galectin-3 on different collagens [38]: they initially found that galectin-3 was strongly upregulated in an animal model of heart failure, and then infused galectin-3 into the pericardial sac of healthy rats to induce left ventricular dysfunction; this led to a 3-fold increase of collagen I over collagen III. A potential mechanism may be that the collagen I promoter, contains a high number of Sp-1 binding sites [39] through which galectin-3 might act [3] but these are not present for collagen III.

MCP also reduced inflammatory responses at day 14 of FA nephropathy which was accompanied by a loss of galectin-3 positive interstitial cells in the kidney. Galectin-3 is upregulated in several other renal diseases with prominent inflammatory components including systemic lupus erythematosus [40], experimental glomerulonephritis [41] and unilateral ureteral obstruction [11], [35]. An alternative name for galectin-3 is Mac-2 as it is abundantly expressed by subsets of macrophages [42]. Galectin-3 can act in an autocrine or parcrine fashion to induce monocyte migration [43] or alternative macrophage activation [7] and regulate cytokine expression [44]. Therefore, as part of this process is mediated by extracellular actions of galectin-3 [9], we propose that MCP effects on inflammation in FA-nephropathy may in part be mediated by galectin-3.

We also observed a small but highly significant reduction in apoptosis with MCP in the later disease phase. This difference is likely to be biologically important because Coles and colleagues [45] previously demonstrated that small changes in measured apoptosis in the kidney actually reflect larger overall changes in cell death due to apoptotic cells being cleared so quickly. Galectin-3 and MCP have different effects on apoptosis: galectin-3 has a BH1 domain of BCL2 that can protect cells from apoptosis [46], [47] and transfection with the lectin makes T-cells resistant to this type of cell death [48]; MCP, in contrast, promotes apoptosis in angiosarcoma [49] and prostate cancer cell lines [31]. This pro-apoptotic effect of MCP is formulation-dependent, however, because alterations in pH and heat-treatment (as we used here to prepare the MCP) can abrogate these pro-apoptotic effects [50]. We suspect that our observed reduction in apoptosis is not directly related to MCP actions on galectin-3 but simply reflects the reduction in disease severity with the modified pectin leading to less remodelling being required; in this case, decreased early proliferation might generate less ‘unwanted’ cells that need to be deleted by apoptosis later.

In conclusion, our data indicates that MCP is protective in experimental nephropathy through modulation of proliferation, apoptosis, fibrosis and inflammation. However, there are two caveats that need to be considered when interpreting this study. Firstly, it is possible that MCP affects other molecular pathways other than galectin-3 in FA nephropathy which need to be investigated further. Secondly, we need to exert caution when interpreting the effect of MCP on galectin-3 levels as these measurements do not account for the bioavailability of the lectin which may be altered. Nevertheless, this study does identify a new potential therapy for acute kidney injury and further experiments are warranted to examine effects of different doses of MCP, timing of treatment and roles in other renal diseases.

## References

[1] Barondes SH, Castronovo V, Cooper DN, Cummings RD, Drickamer K (1994). Galectins: a family of animal β-galactoside binding lectins.. Cell.

[2] Leffler H, Barondes SH (1986). Specificity of binding of three soluble rat lung lectins to substituted and unsubstituted mammalian β-galactosides.. J Biol Chem.

[3] Lin HM, Pestell RG, Raz A, Kim HR (2002). Galectin-3 enhances cyclin D1 promoter activity through SP1 and a cAMP-responsive element in human breast epithelial cells.. Oncogene.

[4] Davidson PJ, Davis MJ, Patterson RJ, Ripoche MA, Poirier F (2002). Shuttling of galectin-3 between the nucleus and cytoplasm.. Glycobiology.

[5] Liu FT, Patterson RJ, Wang JL (2002). Intracellular functions of galectins.. Biochim Biophys Acta.

[6] Nieminen J, Kuno A, Hirabayashi J, Sato S (2007). Visualization of galectin-3 oligomerization on the surface of neutrophils and endothelial cells using fluorescence resonance energy transfer.. J Biol Chem.

[7] MacKinnon AC, Farnworth SL, Hodkinson PS, Henderson NC, Atkinson KM (2008). Regulation of alternative macrophage activation by galectin-3.. J Immunol.

[8] Bullock SL, Johnson TM, Bao Q, Hughes RC, Winyard PJ (2001). Galectin-3 modulates ureteric bud branching in organ culture of the developing mouse kidney.. J Am Soc Nephrol.

[9] Ochieng J, Furtak V, Lukyanov P (2004). Extracellular function of galectin-3.. Glycoconj J.

[10] Winyard PJ, Bao Q, Hughes RC, Woolf AS (1997). Epithelial galectin-3 during human nephrogenesis and childhood cystic diseases.. J Am Soc Nephrol.

[11] Okamura DM, Pasichnyk K, Lopez-Guisa JM, Collins SJ, Hsu DK (2011). Galectin-3 preserves renal tubules and modulates extracellular matrix remodelling in progressive fibrosis.. Am J Physiol Renal Physiol.

[12] Nishiyama J, Kobayashi S, Ishida A, Nakabayashi I, Tajima O (2000). Up-regulation of galectin-3 in acute renal failure of the rat.. Am J Pathol.

[13] Ortiz A, Lorz C, Catalan MP, Danoff TM, Yamasaki Y (2000). Expression of apoptosis regulatory proteins in tubular epithelium stressed in culture or following acute renal failure.. Kidney Int.

[14] Zimmermann HD, Maykemper B, Dieker P (1977). Intra- and extrarenal vascular changes in the acute renal failure of the rat caused by high-dose folic acid injection.. Virchows Arch A Pathol Anat Histol.

[15] Baserga R, Thatcher D, Marzi D (1968). Cell proliferation in mouse kidney after a single injection of folic acid.. Lab Invest.

[16] Long DA, Price KL, Ioffe E, Gannon CM, Gnudi L (2008). Angiopoietin-1 therapy enhances fibrosis and inflammation following folic acid-induced acute renal injury.. Kidney Int.

[17] Long DA, Woolf AS, Suda T, Yuan HT (2001). Increased renal angiopoietin-1 expression in folic-acid induced nephrotoxicity in mice.. J Am Soc Nephrol.

[18] Chiu MG, Johnson TM, Woolf AS, Dahm-Vicker EM, Long DA (2006). Galectin-3 associates with the primary cilium and modulates cyst growth in congenital polycystic kidney disease.. Am J Pathol.

[19] Tsuchiyama Y, Wada J, Zhang H, Morita Y, Hiragushi K (2000). Efficacy of galectins in the amelioration of nephrotoxic serum nephritis in Wistar Kyoto rats.. Kidney Int.

[20] Glinsky VV, Raz A (2009). Modified citrus pectin anti-metastatic properties: one bullet, multiple targets.. Carbohydr Res.

[21] Ridley BL, O’Neil MA, Mohnen D (2001). Pectins: structure, biosynthesis and oligogalacturonide-related signalling.. Phytochemistry.

[22] Gunning AP, Bongaerts RJ, Morris VJ (2009). Recognition of galactan components of pectin by galectin-3.. FASEB J.

[23] Morris VJ, Gromer A, Kirby AR, Bongaerts RJM, Gunning AP (2011). Using AFM and force spectroscopy to determine pectin structure and (bio) functionality.. Food Hydrocolloids.

[24] Nangia-Makker P, Hogan V, Honjo Y, Baccarini S, Tait L (2002). Inhibition of human cancer cell growth and metastasis in nude mice by oral intake of modified citrus pectin.. J Natl Cancer Inst.

[25] Inohara H, Raz A (1994). Effects of natural complex carbohydrate (citrus pectin) on murine melanoma cell properties related to galectin-3 functions.. Glycoconjugate Journal.

[26] Davis B, Dei Cas A, Long DA, White KE, Hayward A (2007). Podocyte-specific expression of angiopoietin-2 causes proteinuria and apoptosis of glomerular endothelia.. J Am Soc Nephrol.

[27] Price KL, Woolf AS, Long DA (2009). Unraveling the genetic landscape of bladder development in mice.. J Urol.

[28] Jang HR, Rabb H (2009). The innate immune response in ischemic acute kidney injury.. Clin Immunol.

[29] Fernandes Bertocchi AP, Campanhole G, Wang PH, Gonçalves GM, Damião MJ (2008). A Role for galectin-3 in renal tissue damage triggered by ischemia and reperfusion injury.. Transpl Int.

[30] Pienta KJ, Naik H, Akhtar A, Yamazaki K, Replogle TS (1995). Inhibition of spontaneous metastasis in a rat prostate cancer model by oral administration of modified citrus pectin.. J Natl Cancer Inst.

[31] Yan J, Katz A (2010). PectaSol-C modified citrus pectin induces apoptosis and inhibition of proliferation in human and mouse androgen-dependent and –independent prostate cancer cells.. Integr Cancer Ther.

[32] Sathisha UV, Jayaram S, Harish Nayaka MA, Dharmesh SM (2007). Inhibition of galectin-3 mediated cellular interactions by pectic polysaccharides from dietary sources.. Glycoconj J.

[33] Baba M, Wada J, Eguchi J, Hashimoto I, Okada T (2005). Galectin-9 inhibits glomerular hypertrophy in db/db diabetic mice via cell-cycle-dependent mechanisms.. J Am Soc Nephrol.

[34] Shimizu M, Khoshnoodi J, Akimoto Y, Kawakami H, Hirano H (2009). Expression of galectin-1, a new component of silt diaphragm, is altered in minimal change nephrotic syndrome.. Lab Invest.

[35] Henderson NC, Mackinnon AC, Farnworth SL, Kipari T, Haslett C (2008). Galectin-3 expression and secretion links macrophages to the promotion of renal fibrosis.. Am J Pathol.

[36] Huang WY, Li ZG, Hus H, Wang X, Jose PA (2009). RGC-32 mediates transforming growth factor-beta-induced epithelial-mesenchymal transition in human renal proximal tubule cells.. J Biol Chem.

[37] Henderson NC, Mackinnon AC, Farnworth SL, Poirier F, Russo FP (2006). Galectin-3 regulates myofibroblast activation and hepatic fibrosis.. Proc Natl Acad Sci USA.

[38] Sharma UC, Pokharel S, van Brakel TJ, van Berlo JH, Cleutjens JP (2004). Galectin-3 marks activated macrophages in failure-prone hypertrophied hearts and contributes to cardiac dysfunction.. Circulation.

[39] Bornstein P, McKay J, Morishima JK, Devarayalu S, Gelinas RE (1987). Regulatory elements in the first intron contribute to transcriptional control of the human alpha 1 (I) collagen gene.. Proc Natl Acad Sci USA.

[40] Kang EH, Moon KC, Lee EY, Lee YJ, Lee EB (2009). Renal expression of galectin-3 in systemic lupus erythematosus patients with nephritis.. Lupus.

[41] Sasaki S, Bao Q, Hughes RC (1999). Galectin-3 modulates rat mesangial cell proliferation and matrix synthesis during experimental glomerulonephritis induced by anti-Thy1.1 antibodies.. J Pathol.

[42] Liu FT, Hsu DK, Zuberi RI, Kuwabara I, Chi EY (1995). Expression and function of galectin-3, a beta-galactoside-binding lectin, in human monocytes and macrophages.. Am J Pathol.

[43] Sano H, Hsu DK, Yu L, Apgar JR, Kuwabara I (2000). Human galectin-3 is a novel chemoattractant for monocytes and macrophages.. J Immunol.

[44] Jeng KC, Frigeri LG, Liu FT (1994). An endogenous lectin, galectin-3 (epsilon BP/Mac-2), potentiates IL-1 production by human monocytes.. Immunol Lett.

[45] Coles HS, Burne JF, Raff MC (1993). Large-scale normal cell death in the developing rat kidney and its reduction by epidermal growth factor.. Development.

[46] Akahani S, Nangia-Makker P, Inohara H, Kim HR, Raz A (1997). Galectin-3: a novel anti-apoptotic molecule with a functional BH1 (NWGR) domain of Bcl-2 family.. Cancer Res.

[47] Fukumori T, Oka N, Takenaka Y, Nangia-Makker P, Elsamman E (2006). Galectin-3 regulates mitochondrial stability and anti-apoptotic function in response to anti-cancer drug in prostate cancer.. Cancer Res.

[48] Yang RY, Hsu DK, Liu FT (1996). Expression of galectin-3 modulates T-cell growth and apoptosis.. Proc Natl Acad Sci USA.

[49] Johnson KD, Glinskii OV, Mossine VV, Turk JR, Mawhinney TP (2007). Galectin-3 as a potential therapeutic target in tumors arising from malignant endothelia.. Neoplasia.

[50] Jackson CL, Dreaden TM, Theobald LK, Tran NM, Beal TL (2007). Pectin induces apoptosis in human prostate cancer cells: correlation of apoptotic function with pectin structure.. Glycobiology.

